# Identification of phlebotomine sand flies (Diptera: Psychodidae) by matrix-assisted laser desorption/ionization time of flight mass spectrometry

**DOI:** 10.1186/1756-3305-7-21

**Published:** 2014-01-14

**Authors:** Vit Dvorak, Petr Halada, Kristyna Hlavackova, Emmanouil Dokianakis, Maria Antoniou, Petr Volf

**Affiliations:** 1 Department of Parasitology, Faculty of Science, Charles University in Prague, Prague, Czech Republic; 2 Laboratory of Molecular Structure Characterization, Institute of Microbiology, Academy of Sciences of the Czech Republic, Prague, Czech Republic; 3 Laboratory of Clinical Bacteriology, Parasitology, Zoonoses and Geographical Medicine, Faculty of Medicine, University of Crete, Crete, Greece

**Keywords:** Species identification, Molecular taxonomy, *Phlebotomus*, MALDI-TOF MS

## Abstract

**Background:**

Phlebotomine sand flies are incriminated in the transmission of several human and veterinary pathogens. To elucidate their role as vectors, proper species identification is crucial. Since traditional morphological determination is based on minute and often dubious characteristics on their head and genitalia, which require certain expertise and may be damaged in the field-collected material, there is a demand for rapid, simple and cost-effective molecular approaches.

**Methods:**

Six laboratory-reared colonies of phlebotomine sand flies belonging to five species and four subgenera (*Phlebotomus*, *Paraphlebotomus*, *Larroussius*, *Adlerius*) were used to evaluate the discriminatory power of matrix-assisted laser desorption/ionization time of flight mass spectrometry (MALDI-TOF MS). Various storage conditions and treatments, including the homogenization in either distilled water or given concentrations of formic acid, were tested on samples of both sexes.

**Results:**

Specimens of all five analysed sand fly species produced informative, reproducible and species-specific protein spectra that enabled their conclusive species identification. The method also distinguished between two *P. sergenti* colonies originating from different geographical localities. Protein profiles within a species were similar for specimens of both sexes. Tested conditions of specimen storage and sample preparation give ground to a standard protocol that is generally applicable on analyzed sand fly specimens.

**Conclusions:**

Species identification of sand flies by MALDI-TOF MS is feasible and represents a novel promising tool to improve biological and epidemiological studies on these medically important insects.

## Background

Phlebotomine sand flies (Diptera: Psychodidae, Phlebotominae) are the only proven vectors of leishmaniases, a group of emerging human and veterinary diseases, which are spreading geographically due to various human activities (migration, landscape and climatic changes) [1]. Leishmaniases are endemic in 98 countries and 3 territories, worldwide [2], putting 350 million people at risk and having a worldwide prevalence of 12 million cases [3]. In addition, sand flies transmit other human pathogens such as bacteria (*Bartonella*) and viruses of families Bunyaviridae, Reoviridae and Rhabdoviridae [1].

Despite their undisputable importance in human and veterinary medicine, inadequate and inconsistent attention has been paid to sand fly species identification. Major taxonomic reviews and identification keys based on morphological characters are now outdated, do not always reflect current views of particular groups and often remain of only regional scope [4]. The conventional approach to sand fly species identification, based on morphological features, requires the mounting of each specimen’s head and abdomen, which bear the decisive characteristics (genitalia, cibarium and pharyngeal armature). Both slide preparation and species identification are laborious and time-consuming, demanding a certain degree of proficiency and expertise. Nevertheless, accurate species identification is profound mainly in epidemiological studies conducted in endemic areas of leishmaniases, where the presence of morphologically similar species with different vectorial capacities can obscure the vector – parasite relationships with consequences in adequate control measures.

In addition to DNA sequencing, various methods of molecular taxonomy have recently been introduced to overcome the shortcomings of traditional sand fly identification based on morphological characteristics, mainly for the purpose of rapid and reliable identification in foci of human leishmaniases. Random Amplified Polymorphic DNA (RAPD) method was deployed to find species-specific DNA profiles of *Phlebotomus papatasi* and *P. duboscqi*, vectors of *Leishmania major* in Africa [5]. Restriction Fragment Length Polymorphism (RFLP) of cytochrome oxidase I gene successfully differentiated between *Phlebotomus riouxi* and *P. chabaudi*, two closely related species of the subgenus *Paraphlebotomus*, suspected vectors of *Leishmania killicki*[6]. Three species of the subgenus *Phlebotomus*, occuring sympatrically in several foci in Sudan and putatively involved in the transmission of *Leishmania major*, were distinguished by a PCR-based assay, amplifying a part of a second internal transcribed spacer [7]. A multiplex species-diagnostic PCR-based assay, based on the 18S rRNA region, was developed in order to rapidly differentiate *P. papatasi* and *P. argentipes*, two species most abundant in the Indian subcontinent [8]. Nevertheless, for routine screening, PCR-based methods of species identification are considered costly, time-consuming and labor intensive. Moreover, their universal applicability is often limited because they target specific sequences. Although they may prove as a suitable solution for a particular taxonomical problem, they can hardly represent a universal tool that would be readily applicable in any situation.

During the last decade protein profiling using the matrix-assisted laser desorption/ionization time of flight mass spectrometry (MALDI-TOF MS) was established as a routine method to identify and classify mainly bacteria for clinical diagnostics [9]. The method is based on acidic extraction of peptides and low molecular weight proteins up to 25 kDa from the studied organism and subsequent MALDI-TOF MS analysis. The peptide/protein mixture obtained by the extraction is mixed with MALDI matrix (typically small aromatic acid) and let to co-crystallize. The crystals are then irradiated by laser pulse and time of flight of the generated ions is measured by mass spectrometer. The recorded mass spectrum serves as a unique protein pattern allowing unambiguous species identification and taxonomical classification. Recently, the approach was also successfully employed for a number of eukaryotic organisms including a panel of 69 *Leishmania* isolates comprising the most important causative agents of human visceral and cutaneous leishmaniases [10]. The concept was proven to be applicable on insects in a pioneering study aimed to distinguish between sibling species of the *Drosophila melanogaster* subgroup [11]. Concerning hematophagous insects, the method is still in its infancy. MALDI-TOF MS was first shown to be applicable on *Culicoides nubeculosus* biting midges [12] and was later deployed in species identification of 15 *Culicoides* species [13,14], five tsetse fly species [15] and a number of mosquito species [16,17].

The aim of this study was to show, for the first time, the applicability of MALDI-TOF MS to identify and distinguish phlebotomine sand fly species, of both sexes, under different conditions of storage and homogenization and to test its discriminatory power regarding subgenera, species and populations. The discriminatory power of the used MS-based approach was tested on five Mediterranean species, proven vectors of important *Leishmania* parasites, namely: *L major* (*P. papatasi*), *L. tropica* (*P. sergenti* and *P. arabicus*) and *L. infantum* (*P. perniciosus* and *P. tobbi*).

## Methods

### Insects

The study was carried out using sand fly specimens reared in laboratory colonies maintained at standard conditions [18] in the insectary of the Department of Parasitology, Charles University in Prague. Specimens from six colonies of five different species were analyzed (country of origin of females used to establish the colony is given in brackets): *Phlebotomus* (*Phlebotomus*) *papatasi* (Turkey), *P.* (*Paraphlebotomus*) *sergenti* (Turkey, Israel), *P.* (*Larroussius*) *perniciosus* (Spain), *P.* (*Larroussius*) *tobbi* (Turkey), *P.* (*Adlerius*) *arabicus* (Israel). Individuals were taken from pools homogenous in age, kept under standard conditions, given the same diet.

### Preparation of samples for MALDI-TOF MS

Insect bodies, stored at various conditions, were dried at room temperature and dissected, cutting off the head and abdomen so that body parts bearing decisive characters could be mounted on slides for morphological analysis, the rest of the abdomen was spared for DNA isolation. Remaining thoraxes were manually ground in 1.5-mL microtubes with 10 μl of homogenization solution using disposable pellets and pestles. Two homogenization solutions were tested: sterile distilled water and 25% formic acid.

### MALDI-TOF MS analysis and spectra evaluation

Two μl of the ethanol or the water protein extract were mixed with 2 μl of a MALDI matrix in a tube. One μl of the resulting mixture was deposited on the MALDI target and allowed to air-dry. The MALDI matrix was prepared daily as an aqueous 60% acetonitile/0.3% TFA solution of sinapinic acid (30 mg/ml; Sigma). Positive-ion mass spectra were measured in linear mode on an Ultraflex III MALDI-TOF spectrometer (Bruker Daltonics, Bremen, Germany) within a mass range of 2–25 kDa and calibrated externally using the Bruker Protein Calibration Standard I. Each acquired spectrum corresponded to an accumulation of 1000 laser shots (5×200 laser shots from different positions of the target spot). The spectra were exported to the MALDI Biotyper 3.1 software for data processing (normalization, smoothing, baseline subtraction, peak picking) and evaluation by cluster analysis. Only a maximum of 100 peaks with signal-to-noise ratio of >3 and relative intensity of at least 0.1% of the most intense peak from the spectra were considered for choosing peaks. For MSP dendrogram creation, an individual main spectrum was generated from each of the acquired spectra.

## Results

The 300 insects used in the analyses were stored either dry-frozen at −20°C or in ethanol of different grades and concentration (denaturized or molecular biology-grade ethanol; 70% or 96% concentration). Results obtained showed that the specimens kept frozen provided the best spectra in terms of peak number, signal intensity, peak resolution, and signal-to-noise ratio (data not shown). However, it is not always possible to freeze sand fly bodies in the field; therefore, storage in ethanol is often the only choice. The use of molecular biology-grade ethanol was more favourable since the specimens kept in denaturized ethanol produced rather noisy spectra with an enhanced baseline. Regarding the ethanol concentration, using 70% ethanol (standard preservation protocol) resulted in reproducible and high quality protein profiles whereas the spectra of insects kept in 96% ethanol were poor with low number of peaks, probably because of inhomogeneous crystallization due to higher content of organic solvent.

The homogenization of the sand flies in two different solutions, sterile distilled water and 25% formic acid, was also tested. Water gave superior results for the frozen individuals, whereas the specimens stored in ethanol exhibited spectra of lower quality and reproducibility. On the contrary, stable and consistent protein profiles were always obtained for individuals stored in 70% ethanol and homogenized in 25% formic acid (data not shown). Considering these observations, we chose the following as our working procedure of the sample preparation: storage of sand fly specimens in molecular biology-grade 70% ethanol and homogenization of the insect body in 10 μl of 25% formic acid. Based on the previous work on *Culicoides* biting midges [12] an aqueous solution of sinapinic acid (30 mg/ml) in 60% acetonitile/0.3% TFA was adopted as MALDI matrix.

**Figure 1:**
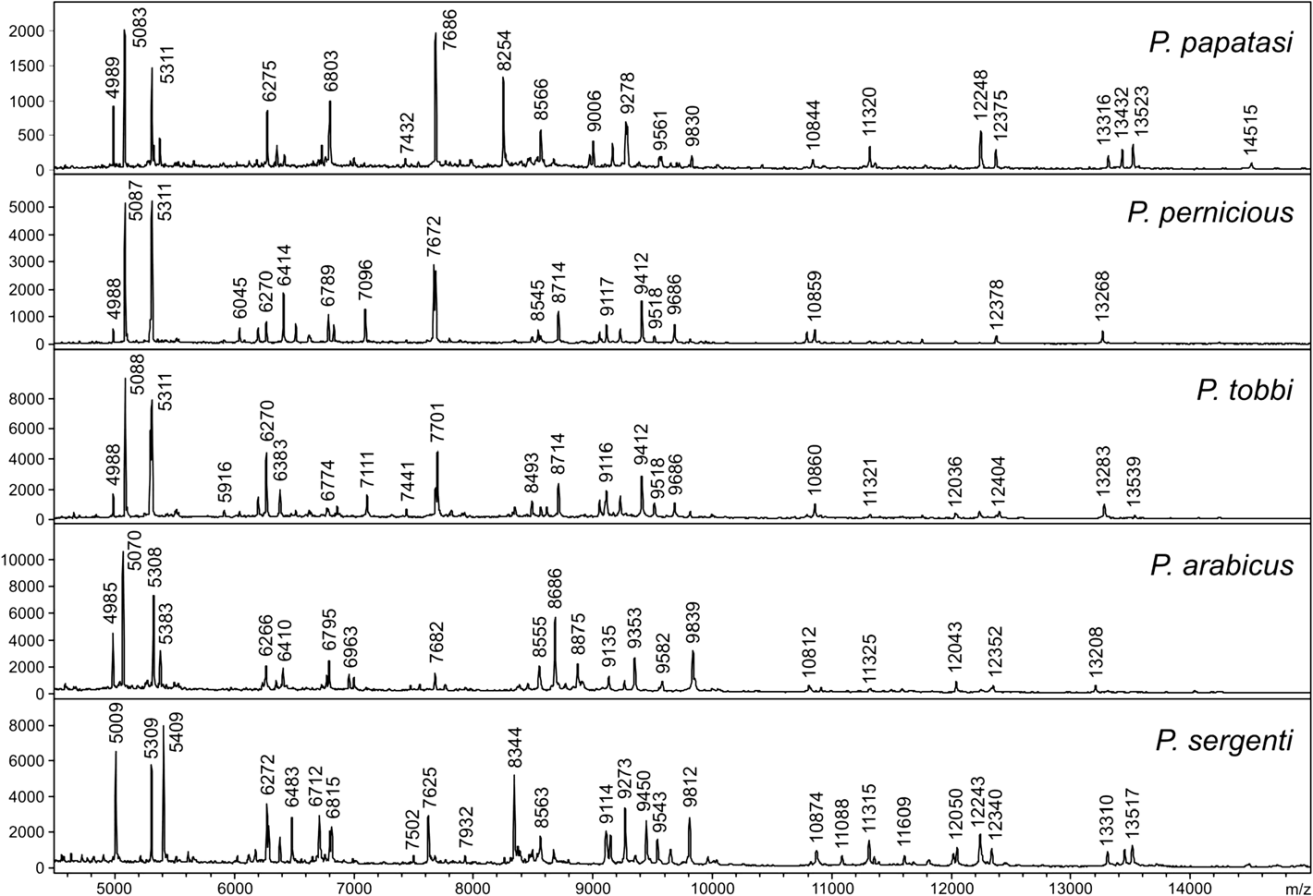
**MALDI-TOF MS protein profiles of five different species of the genus *Phlebotomus *showing species-unique peaks for conclusive species identification.** The peaks in the mass spectra represent peptides or small proteins obtained from the sand fly bodies using acidic extraction.

**Figure 2:**
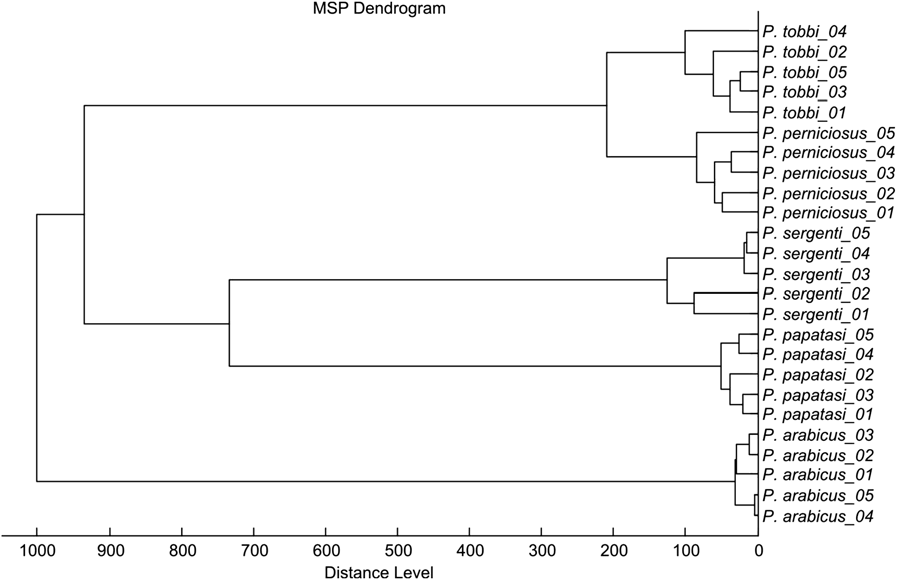
**Dendrogram obtained by cluster analysis of MALDI-TOF MS spectra of five *Phlebotomus *representatives.** Five female individuals of each species were analyzed. Distance is displayed in relative units.

**Figure 3:**
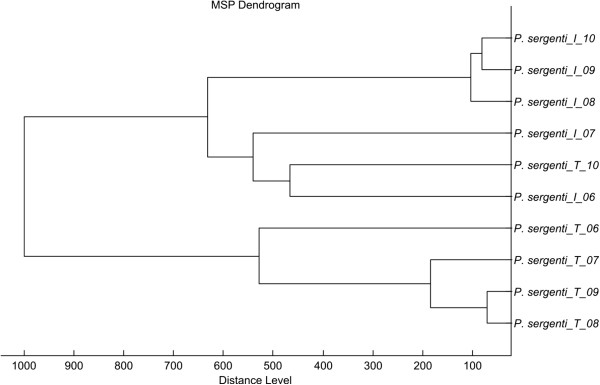
**Dendrogram obtained by cluster analysis of MALDI-TOF MS spectra of specimens of *Phlebotomus sergenti *from laboratory colonies originating from Turkey and Israel.** Distance is displayed in relative units.

Using the above conditions, all five *Phlebotomus* species tested generated reproducible protein spectra with a high number of intense signals within the mass range of 2–25 kDa (Figure 1). The protein profiles obtained were species-specific with several species-unique peaks that allowed reliable and conclusive species identification and taxonomical classification of the analyzed sand flies as shown by hierarchical cluster analysis (Figure 2). The method recognized all but two specimens of *P. sergenti* of laboratory colonies originating from two geographically distant regions (Figure 3).

**Figure 4:**
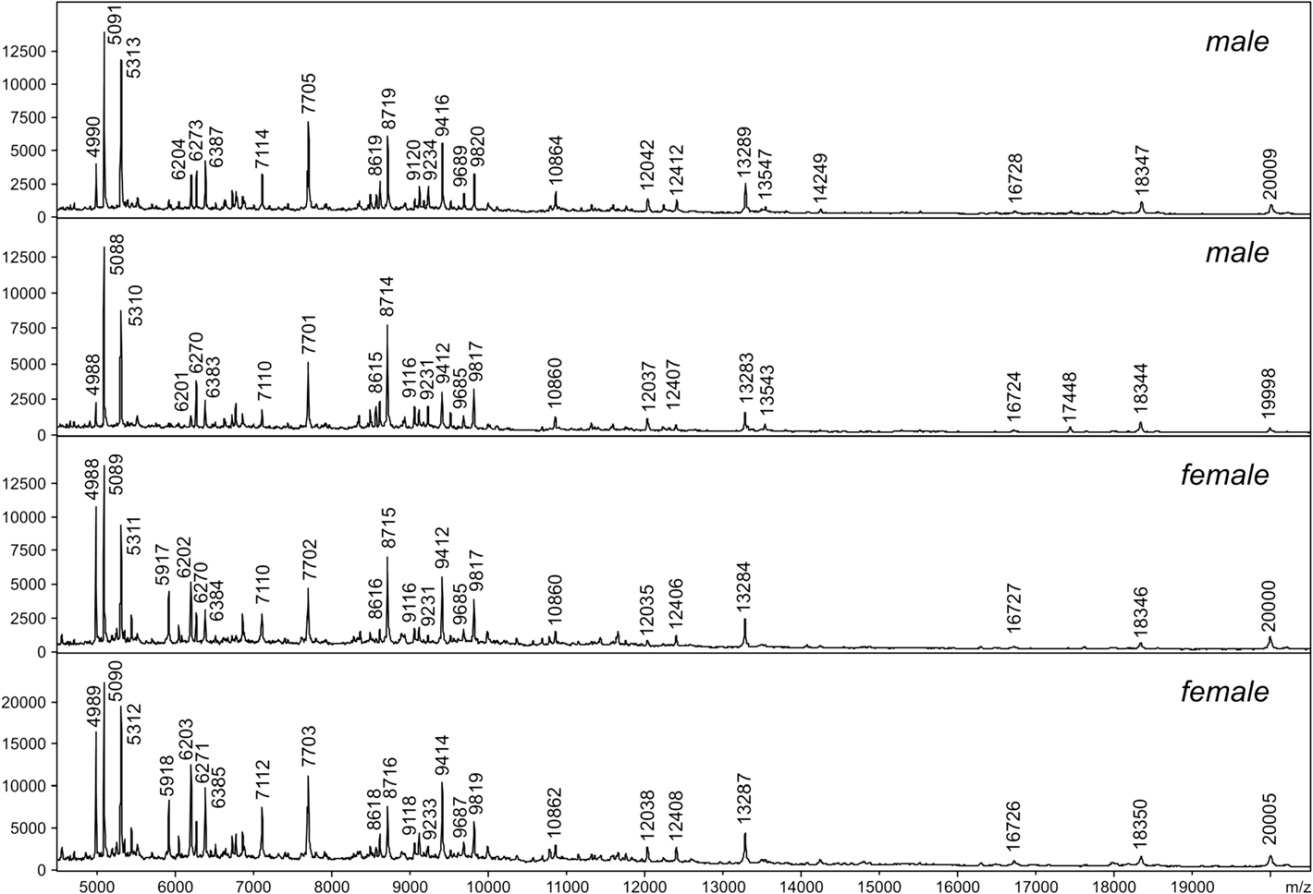
**Comparison of MALDI-TOF MS spectra of two males and two females of *Phlebotomus tobbi*.** Nearly identical spectra were observed for both sexes with all species-specific peaks present.

**Figure 5:**
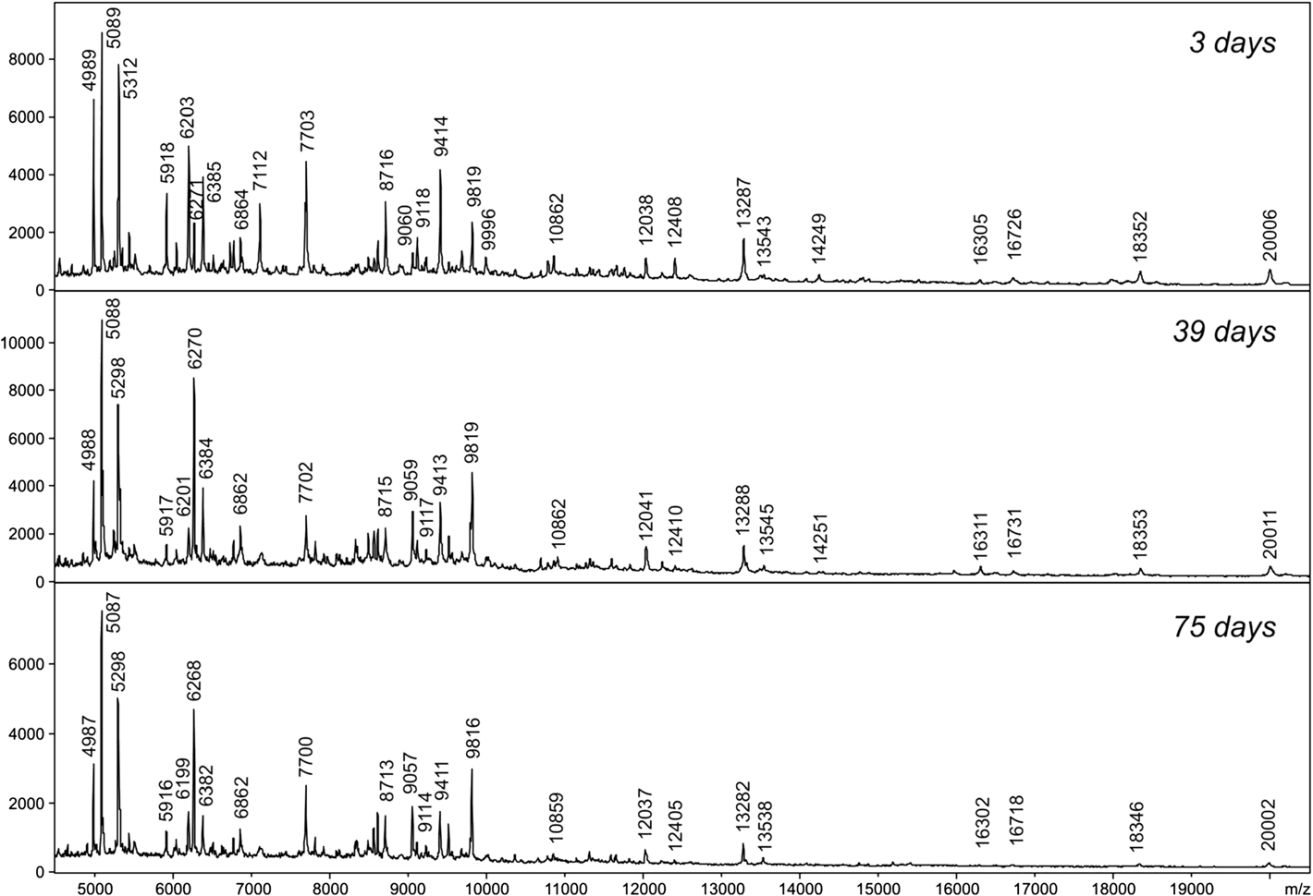
**Effect of the storage conditions on the spectra quality.** Comparison of MALDI-TOF MS spectra of *Phlebotomus tobbi* females stored in 70% ethanol for 3, 39 and 75 days.

**Figure 6:**
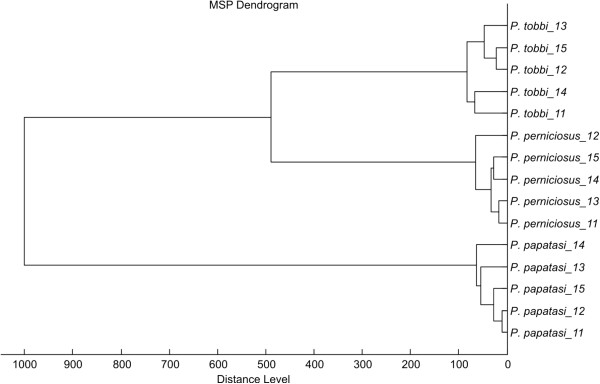
**Species clustering observed for five female individuals of three *Phlebotomus *species after storage in 70% ethanol for 75 days.** Distance is displayed in relative units.

For all tested *Phlebotomus* species, an equal number of males and unfed females were analysed. The data obtained from male and female individuals displayed very similar spectrum patterns and most of the major protein peaks were identical to both genders (Figure 4). To investigate the influence of storage time, protein spectra of specimens kept in 70% ethanol for the period of 3, 39 and 75 days were compared (Figure 5). The protein profiles for all storage length conditions were reproducible and enabled unambiguous species differentiation even after longer periods of storage in ethanol (Figure 6).

## Discussion

During the last decade, protein profiling by MALDI-TOF MS has been exploited mainly for the identification of unicellular pathogens. It became an analytical tool that offers high-throughput, sensitive and specific analysis for applications in microbiology, including demanding clinical diagnostics in terms of reproducibility as well as cost-effectiveness [9,19]. The method offers sufficient discriminatory power for a wide range of clinically relevant bacteria not only at the species level, but also at the subspecies and strain levels, allowing the detection of epidemic lineages. The vast amount of accumulated data led to the establishment of several commercial and non-commercial spectra databanks that are at the disposal for routine microbiological assays [20].

Only recently researchers ventured into applications of this method on multicellular organisms including medically important insects. It was first evaluated on a model species of the biting midge *Culicoides nubeulosus* under various conditions of sample storage and preparation [12]. A reference database of biomarker mass sets was then established for 15 species of biting midges to enable their automated database-based identification [13]. The approach successfully distinguished between two morphologically unrecognisable cryptic species within *Culicoides grisescens*. Subsequently, the database proved to be useful for large-scale species identification in a three-year entomological survey of *Culicoides* biting midges in different climatic regions of Switzerland, being the first application of this method in a field study [14]. When applied to 34 laboratory colonies and wild-caught specimens of 12 *Anopheles* species, MALDI-TOF MS protein profiling accurately identified even closely related cryptic species within the *A. gambiae* complex [16]. It also accurately identified 20 mosquito species of six genera upon the profiles of leg protein extracts [17].

Sand flies, in several aspects, are similar to biting midges: small body size, minute species-specific morphological features of high epidemiological significance; which make them ideal candidates for alternative approaches towards taxonomical identification. Their role as exclusive vectors of *Leishmania*, a group of medically and veterinary important kinetoplastid parasites, urges the need for accurate species identification that is essential for effective control measures and yet not always easy to achieve. The advent of molecular approaches has already revealed the existence of several species complexes in sand flies, which harbour morphologically undistinguishable cryptic species. Within the *Phlebotomus perniciosus* complex, a new and yet formally undescribed sibling species of *P. longicuspis* was found [21]. *Phlebotomus argentipes*, the main vector of *Leishmania donovani* in the Indian subcontinent, is a complex of at least three species of challenging morphology and unknown role in the transmission of the disease [22]. *Phlebotomus major* complex comprises of a number of closely related species that overlap geographically in some areas in the Mediterranean Basin [23]. Different DNA-based methods were deployed in the study of these species complexes, a fact emphasizing the need for a more universal tool.

By analysing more than 300 individuals belonging to five medically important sand fly species of the genus *Phlebotomus*, all vectors of different *Leishmania* species infecting humans, we demonstrated that protein profiling using MALDI-TOF MS can be used for rapid and reliable species identification of phlebotomine sand flies. All analysed species produced distinct, consistent and reproducible species-specific protein spectra, which were constructive in species identification. There were no differences between males and unfed females of the respective species that would obscure their identification. In our method of body utilization of specimens the terminal body parts were not included in the acidic extraction prior to MALDI-TOF MS, which possibly removed some of the sex-specific proteins. This is in agreement with the previous findings where both sexes of the same species were analysed. In the former study on *Drosophilla* species, a biological meaning of the peaks detected by MALDI-TOF MS protein profiling was investigated by nano-HPLC electrospray ionisation tandem mass spectrometry. Most of them were identified as originating from muscle tissues and mitochondria and are, therefore, probably identical for both sexes [24].

Comparing different sand fly storage methods revealed that the best results are obtained if specimens are preserved dry-frozen. However, in the field, it is not always possible to freeze freshly caught sand flies. Therefore, the fact that even long-term sample storage in 70% ethanol provides satisfactory and reproducible results is very promising given that ethanol is the usual medium for storing samples for entomological surveys and for processing by molecular techniques. Sand fly females engorged with host blood were not tested in this initial study as it is known from studies on hematophagous biting midges that the presence of blood has a considerable impact on MALDI-TOF patterns, reducing the intensity of the biomarker masses [12]. To study the influence of bloodmeal presence at different digestion stages on the protein patterns, remains one of the tasks in future studies.

One of the aims of this study was to evaluate the levels of discriminative power of MALDI-TOF MS protein profiling. So far, in some studies it failed to differentiate between geographical populations of tested species in some studies, namely closely related *Drosophila* species [24] and individuals of three *Culicoides* species collected at either site of the Alpine crest and exhibited similar protein profiles [13]. On the contrary, when tools of linear discriminant analysis were deployed in a study on anopheline mosquitoes, MALDI-TOF MS resolved even colony-specific patterns [16]. In our study, it was possible to distinguish between all but two analyzed specimens of *Phlebotomus sergenti* from two laboratory colonies originating from Turkey and Israel. Based on the sequencing analysis of internal transcribed spacer (ITS), it was previously postulated that these populations represent two distinct lineages that may constitute cryptic species within *P. sergenti*[25], although this hypothesis was later not confirmed by analyses of other genes [26,27]. Nevertheless, specimens from actual wild populations should be analyzed by MALDI-TOF MS to further evaluate our promising findings on the ability of this method to differentiate between populations of geographically different origin.

Our findings suggest that MALDI-TOF MS-based species characterization may represent a rapid, simple, reproducible and also cost-effective alternative. It was estimated, roughly, that one MALDI-TOF MS assay can be 100 times less expensive than a PCR run, not including labour costs and processing time. While the acquisition of the machine itself represents a major investment, its use thereafter is cost-effective [16]. Moreover, the described workflow enables the utilization of the same sand fly specimen in morphological analysis, PCR-based assay (sequencing etc.) and protein profiling by MALDI-TOF MS. Therefore, the method clearly has a potential to become an invaluable complementary tool to established approaches towards sand fly species identification and taxonomical classification.

## Conclusions

The present study shows that protein profiling by MALDI-TOF MS is applicable on phlebotomine sand flies and represents the first step towards the establishment of a protein spectra database that would enable quick and reliable species identification, a much desired tool for many applications in vector biology and epidemiology of the leishmaniases.

## Competing interest

The authors declare that they have no competing interests.

## Authors’ contributions

VD, PV, MA and ED designed the study. PH, VD and KH carried out laboratory experiments and PH conducted data analysis. All authors contributed to the manuscript and approved the final version of the manuscript.
